# Chemical Composition, Antioxidant, Enzyme Inhibition and Anticancer Activities: Effects on the Expression of Genes Related to Apoptosis and the Polyamine Pathway and Molecular Docking Analyses of *Rhus coriaria* L. Extracts

**DOI:** 10.3390/cimb48010010

**Published:** 2025-12-22

**Authors:** Canan Yılmaz-Kapancık, Burak Tüzün

**Affiliations:** 1 Department of Biochemistry, Faculty of Pharmacy, Anadolu University, 26470 Eskişehir, Turkey; 2 Plant and Animal Production Department, Technical Sciences Vocational School of Sivas, Sivas Cumhuriyet University, 58140 Sivas, Turkey

**Keywords:** antioxidant, anticancer, apoptosis, polyamine pathway, enzyme inhibition, molecular docking, *Rhus coriaria* L.

## Abstract

DNA damage resulting from oxidative stress plays a major role in cancer formation. Despite DNA damage, the inability of cells to enter apoptosis due to irregularities in apoptotic protein levels and the induction of their proliferation as a result of the increase in polyamine levels causes the development and progression of cancer. The anticancer effects of *Rhus coriaria* L. extracts on lung cancer, colon cancer and fibroblast cell lines were determined by MTT (3-(4,5-Dimethylthiazol-2-yl)-2,5-Diphenyltetrazolium Bromide). Total antioxidant status (TAS) was analyzed with a commercial kit. The expression levels of genes related to apoptosis and the polyamine pathway in lung and colon cancer cell lines were analyzed by a Real-time Polymerase Chain Reaction (RT-PCR) device. *Rhus coriaria* L. extracts were found to have anticancer effects selectively on A549 and HT-29 cancer lines. It has also been shown that *Rhus coriaria* L. extracts have strong antioxidant capacity and can inhibit the Xanthine Oxidase (XO) enzyme in a dose-dependent manner. Afterwards, the interactions of the molecules in extracts of *Rhus coriaria* L. against various proteins such as colon cancer protein (PDB ID: 3DTC and 4UYA) lung cancer protein (PDB ID: 4ZXT and 5ZMA) were examined, and their activities were compared. MM/GBSA methods of the molecule with the best docking score are calculated as binding free energy.

## 1. Introduction

Scientific studies to determine and develop the medical benefits of plants have been increasing in recent years. Medicinal plants are used to protect human health and treat diseases. Medicines used to treat diseases first consisted only of plants. Over time, the active substances of plants that treat diseases began to be isolated and produced synthetically and became the raw materials of the pharmaceutical industry [1,2]. Medicinal plants are also of great importance in the treatment of cancer. Chemotherapy or chemotherapy combinations are used in today’s cancer treatment. As is known, these treatment methods have disadvantages called side effects. Medicinal plants are used to eliminate these side effects [3].

More reactive oxygen species production than antioxidant capacity causes oxidative stress in cells. Increased oxidative stress supports the formation of carcinogens by damaging the building blocks of cells such as proteins and lipids, especially DNA. In the presence of oxidative stress, inflammation occurs in tissues due to cellular damage. In normal cases, damage in cells harmed by inflammation is repaired or cells are replaced. However, in cases of consecutive inflammation, this is not possible, and a chronic inflammation process occurs. In this inflammation process, the induction of synthesis of growth factors, proinflammatory cytokines and cyclooxygenase enzymes causes changes that support tumor formation. It is possible to prevent oxidative stress and therefore inflammation by increasing antioxidant capacity. Therefore, increasing antioxidant capacity is of great importance in preventing cancer [4,5,6,7].

Maintaining cellular balance in the organism is possible through apoptosis, which is programmed cell death. In the emergence and continuation of cancer, variations in apoptotic pathways cause the balance to shift towards cell proliferation, along with a decrease in apoptosis. In the intrinsic pathway of apoptosis, after DNA damage caused by oxidative stress along with external stimuli, apoptosis is induced by mediators including BAX and BCL-2 proteins. However, the Caspase-3 enzyme mediates the occurrence of apoptosis by causing cellular proteins to be cut and disintegrated in the last stage of apoptosis [8]. However, in cancer, as there are variations in the levels of proteins and enzymes related to apoptosis, including BAX, BCL-2 and Caspase-3 on the apoptotic pathway, apoptosis is inactivated and the rapid progression of cancer continues [8,9].

Spermine, spermidine and putrescine, which constitute the polyamine class, are polycationic alkylamines found in cells at millimolar concentrations. Despite their low molecular weight, they have many vital functions in cells. Their most important cellular functions are the stabilization of chromatin structure and healthy maintenance of protein synthesis, apoptosis, regulation of ion channels, and roles in cell growth, differentiation and survival. Cells absolutely need polyamines to survive and proliferate. Polyamine levels vary in cancer. The reason for this is that cancer cells need high polyamine levels to proliferate rapidly. Ornithine is a precursor in the synthesis of polyamines. It is used in the synthesis of polyamines via ornithine decarboxylase (ODC). In addition, agmatine is synthesized from arginine by the enzyme arginine decarboxylase (ADC). Polyamines are synthesized from agmatine by the enzyme agmatinase (AGMAT). Since cancer cells need high polyamine usage, polyamine synthesis is increased by changing the enzyme levels in the polyamine pathway [10,11,12].

*Rhus coriaria* L. plant is grown in the Mediterranean region. *Rhus coriaria* L. is especially rich in flavonoids and polyphenolic compounds, so it is used in the treatment of many diseases, including cancer and cardiovascular diseases [13,14]. It has also been shown to have anticancer activity on cancer cell lines [15]. Additionally, ethanol extract has been shown to have the ability to reverse damage in rat retinal neuron cells and thus has neuroprotective properties [16].

Plants have been considered an important natural resource used in the treatment of various diseases for thousands of years [17]. Plant extracts contain a mixture of biologically active components derived from plants, and these components have a broad spectrum of pharmacological activity. Modern science has developed advanced techniques and methods to investigate the potential therapeutic effects of herbal compounds. Molecular docking simulation has emerged as a critical and important tool to understand the interactions of plant extracts with biomolecular targets [18].

Molecular docking is a computational technique used to predict how small molecules bind to active sites of biomolecular targets, such as proteins. This method allows assessing how a ligand (e.g., a compound found in a plant extract) interacts with a specific protein target and the binding affinity of this interaction [19]. Docking scores indicate how strongly the ligand binds with the target protein, and these scores are used as an indicator of potential biological activity.

In recent years, the integration of molecular docking simulation has become increasingly important in order to understand the biological activities of plant extracts and use them in the drug discovery process [20]. These techniques offer the opportunity to more quickly and efficiently evaluate the interactions of herbal compounds with target proteins and their potential therapeutic effects. Additionally, these calculations provide guidance for experimental studies, helping researchers identify the most promising candidates and optimize their research processes [21].

The most widely known aspect of them is the fact that molecular docking calculations are an essential component of the process of creating and discovering novel medications. Predicting the binding and interaction patterns of prospective compounds with a target protein is part of the process of evaluating the efficacy and selectivity of proposed medications [22]. It has been demonstrated through prior studies that the implementation of computer environments early on results in a decrease in the expenses and the amount of time needed for laboratory testing. In addition, the interactions that occur between drugs and other small molecules and proteins are understood by way of molecular docking simulations [23]. This provides us with insight into how specific illnesses interact with pharmaceuticals and how these medications function, both of which contribute to the treatment of illness. Therefore, the relationship between the major active molecules in *Rhus coriaria* L. and proteins associated with lung and colon cancer was investigated through molecular docking simulations and was intended to be evaluated together with the experimental data obtained.

In our study, extract contents of *Rhus coriaria* L. were analyzed using GC-MS. However, the total antioxidant content was determined by means of a commercial kit. The effect of plant fruit on the expressions of apoptosis and polyamine pathway genes in A549 and HT-29 was determined by RT-PCR. The relationship between active substances in plant fruit and proteins in lung and colon cancer was analyzed by the molecular docking method. Afterwards, the interactions of the molecules in extracts of *Rhus coriaria* L. against various proteins such as colon cancer protein (PDB ID: 3DTC and 4UYA) [24,25] and lung cancer protein (PDB ID: 4ZXT and 5ZMA) [26,27] were examined, and their activities were compared. MM/GBSA methods of the molecule with the best docking score are calculated as binding free energy.

## 2. Materials and Methods

### 2.1. Plant Material

*Rhus coriaria* L. fruit was harvested from the southern Anatolia region of Turkey in 2023 and stored in a dark environment at room temperature.

### 2.2. Extract Preparation

Methanol, water and chloroform extracts were prepared by grinding 10 g of the collected plant sample. For the preparation of methanol extract, plant samples were extracted at 60 °C for 2 h in a Soxhlet extractor containing methanol. The extract was concentrated at 45 °C and under vacuum. The extracts were suspended in water, and less-polar molecules were separated with chloroform. After the water-soluble and water-insoluble fractions were evaporated, the residue was stored in the dark at 4 °C.

### 2.3. Gas Chromatography-Mass Spectroscopic Analysis

In GC-MS analyses, a Shimadzu GCMS-QP2010 Ultra Gas Chromatography Mass Spectrometer (Shimadzu, Kyoto, Japan) and ultra-narrow capillary columns were used. The system setup is equipped with a Flex 2 autosampler (EST Analytical, West Chester Township, OH, USA) for automatic HS-SPME sampling. Analyses of the obtained data were carried out through GCMS Postrun Analysis software (ver. 4.53).

### 2.4. Cell Culture Maintenance

#### 2.4.1. Preparation of Cell Culture

A549, HT-22 and L929 mycoplasma-free cell lines were provided by ATCC. A549 lung cancer, HT-29 colon cancer and L929 fibroblast cells were grown in medium (containing Dulbecco’s Modified Eagle Medium (DMEM)) containing 10% of the total medium volume with Fetal Bovine Serum (FBS) and 1% penicillin-streptomycin at 37 °C in 5% CO_2_ supplemented with DMEM. Cells were proliferated at 37 °C in 5% CO_2_ [28].

#### 2.4.2. MTT Method

With the help of the MTT method, IC_50_ values were determined in A549, HT-29 and L929 cells exposed to methanol, water and chloroform extracts of *Rhus coriaria* L. for 24, 48 and 72 h. The extracts were prepared by dissolving them in dimethyl sulfoxide (DMSO). During the application of the extracts, the cells were exposed to DMSO at a concentration lower than 0.1%, which is the concentration at which no toxic effects were observed. Cells were incubated overnight in 96-well plates at 1 × 10^5^/mL in 100 μL of medium. Then, % viability values were determined as given in the kit protocol [29]. From the data obtained, % viability graphs were drawn using the GraphPad Prism (version 10) method, and statistical analysis of the data was performed.

### 2.5. Expression Analysis of Genes Associated with Apoptosis and the Polyamine Pathway

Total RNA was isolated from A549 and HT-29 cells exposed to IC50 dose of chloroform extracts of *Rhus coriaria* L. using a commercial kit (GeneAll Hybrid-R, Seoul, Republic of Korea). Then, cDNA was synthesized using a commercial kit (Thermo Scientific, Waltham, MA, USA) in accordance with the kit protocols. Expression analyses of genes associated with apoptosis and polyamine pathway were performed using the SYBR Green qPCR Mastermix kit on the RT-PCR device (Thermo Scientific, Waltham, MA, USA). Statistical analysis of the data was carried out with the ΔΔCT method with the help of the https://dataanaliz2.qiagen.com/pcr program (accessed on 15 December 2025) [30].

### 2.6. XO Enzyme Inhibition Measurement

XO inhibitory activity was determined using 96-well plates under aerobic conditions using the procedure reported by Noro et al., with appropriate modifications using a spectrophotometer (BMG Labtech, Cary, NC, USA) [31].

### 2.7. TAS Measurement

TAS was determined using a commercial kit (Rel Assay Diagnostic, Şehitkamil, Turkey) and in accordance with the kit protocols [32].

### 2.8. Theoretical Methods

Schrödinger developed the Maestro Molecular Modelling platform (version 12.8), which was used to carry out the molecular docking calculations [33]. There were several steps involved in the procedure. To examine the interactions that exist between the molecules and the protein after the latter has undergone preparation, the LigPrep module [34], the Glide ligand docking module [35,36] and the protein preparation module [37] were employed in the first phase. The OPLS4 method was used to do all of the calculations. In addition, the potential of the compounds that were being examined as drugs was assessed using absorption, distribution, metabolism, excretion and toxicity (ADME/T) tests. The effects and reactions of chemicals in human metabolism were predicted by utilizing the Qik-prop module [38] of the Schrödinger software (version 2022-4).

Molecular docking calculations were performed to represent physiological conditions. All protein and ligand preparation steps were carried out under neutral pressure conditions (1 atm), and no high-pressure or variable-pressure modes were applied in the calculations. Protein and ligand models were prepared under physiological pH conditions (pH = 7.0 ± 0.2) to mimic biological systems. In this context, the protonation states of the protein structures were optimized at pH 7.0 using the Protein Preparation Wizard module; ionizable amino acid residues and terminal groups were assigned to appropriate protonation states. Ligand structures were protonated in the same pH range using the LigPrep tool, and possible ionic and tautomeric forms were included in the docking calculations. This approach enabled the modeling of protein–ligand interactions in a manner closest to physiological conditions and increased the biological significance of the obtained docking results.

### 2.9. MM-GBSA Calculation

The binding free energy of the complexes between the ligand and the protein was calculated by employing the MM-GBSA technique, which is included in the Schrödinger Prime module (Schrodinger, 2021a) [39]. By default, various options were configured. The OPLS4 force field, VSGB solvent model and rotamer search methods were employed in the process of establishing the binding free energy [40]. We utilized the following equation in this case in order to determine the binding free energy of each and every complex: ΔG_bind = G_complex − (G_protein + G_ligand).

Where G_ligand is the ligand’s free energy value, G_protein is the target protein’s free energy value, G_complex ligand–protein complexes are the free energy value and ΔG_bind is the binding free energy.

## 3. Results and Discussion

### 3.1. Chemical Composition

GC-MS analysis was performed for the chloroform extract with the highest anticancer activity. Retention times and % areas of the components of the chloroform extract were determined (Table 1). It was determined that there were 41 active compounds in the highest concentration in the chloroform extract.

In the chloroform extract of *Rhus coriaria* L., Phenol, 3-pentadecyl-, 1-heptacosanol, 9-octadecenamide and hexadecanoic acid were the active constituents (Table 1).

### 3.2. %Cell Viability Assay

**Figure 1:**
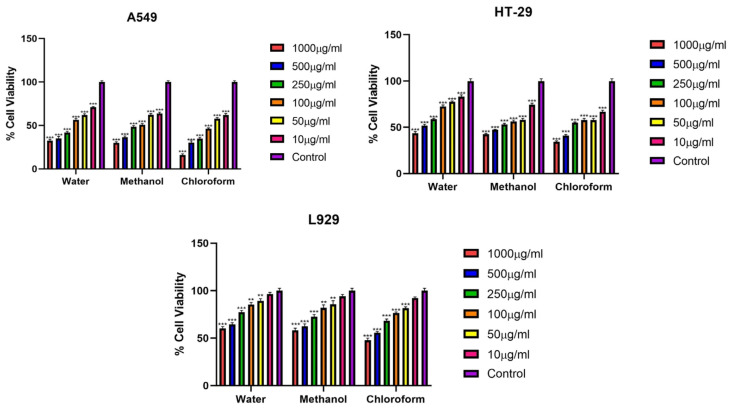
Viability percentages of A549, HT-29 and L929 cells after 24 h exposure (*** *p* < 0.001, ** *p* < 0.01). Error bars represent standard deviation.

**Figure 2:**
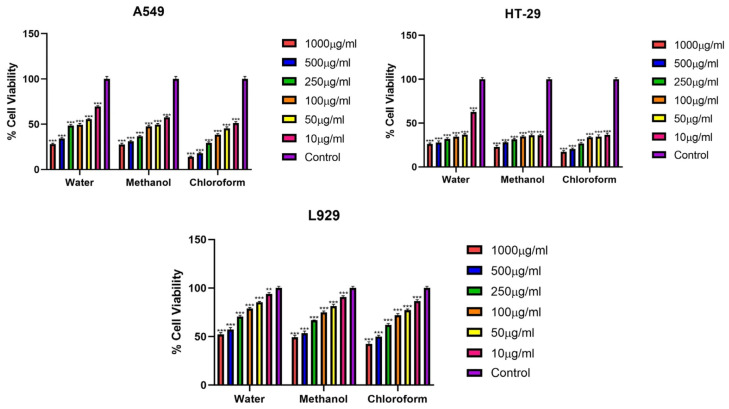
Viability percentages of A549, HT-29 and L929 cells after 48 h exposure (*** *p* < 0.001, ** *p* < 0.01). Error bars represent standard deviation.

**Figure 3:**
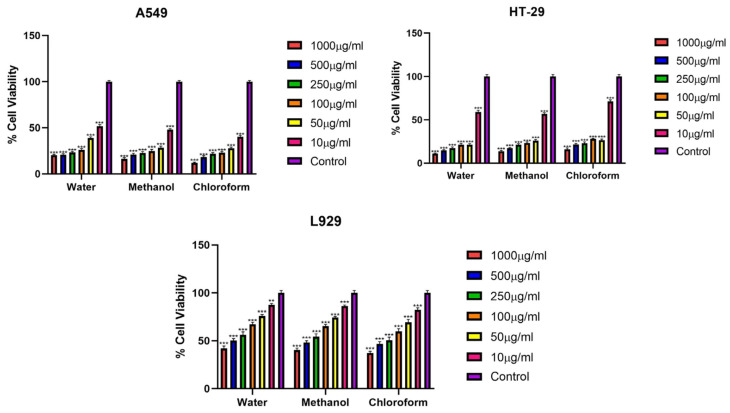
Viability percentages of A549 and HT-29 after 72 h incubation (*** *p* < 0.001, ** *p* < 0.01). Error bars represent standard deviation.

The cytotoxic effects of fractions of *Rhus coriaria* L. plant extracts obtained using different solvents (water, methanol and chloroform) on lung cancer cell line A549, colon cancer cell line HT-29 and normal fibroblast L929 cell line were investigated by MTT method at 24, 48 and 72 h (Figure 1, Figure 2 and Figure 3) (Table 2, Table 3 and Table 4).

Based on IC_50_ values at 24 h, it was determined that the extracts exhibited varying levels of cytotoxic effects against the cell lines. The chloroform extract exhibited the strongest cytotoxic effect on A549 and HT-29 cell lines. The methanol extract had moderate cytotoxicity on these cell lines, and the water extract had the weakest. However, all plant extracts had very high IC_50_ values on the normal fibroblast cell line L929. This suggests that the extracts exhibit selective anticancer activity against cancer cells. The chloroform extract, in particular, exhibited high cytotoxic activity against cancer cells and low toxicity against normal cells, indicating that this extract has high selective anticancer potential (Figure 1) (Table 2).

At 48 h, all extracts exhibited varying levels of cytotoxicity against the cell lines. At 24 h, the chloroform extract exhibited the highest cytotoxicity against both A549 and HT-29 cancer cells. The methanol extract also exhibited a similarly high level of cytotoxicity to the chloroform extract, while the water extract had a lower cytotoxicity. In the normal fibroblast cell line L929, IC_50_ values of all extracts were found to be quite high. These results demonstrated that the methanol and chloroform extracts had potent cytotoxic activity against A549 and HT-29 cancer cells while exhibiting limited toxicity in normal cells (Figure 2) (Table 3).

At 72 h, the extracts were found to exhibit a time- and dose-dependent increase in cytotoxicity in all cell lines. IC_50_ values were found to decrease significantly after 72 h. The chloroform extract again caused the strongest cytotoxic effect compared to the other extracts. Subsequently, the methanol extract showed a high level of cytotoxicity, while the water extract showed a lower cytotoxicity. The high IC_50_ values on the normal fibroblast cell line L929 suggest that the extracts exhibit selective anticancer effects against cancer cells. This selectivity in anticancer activity was particularly pronounced in the chloroform and methanol extracts (Figure 3), (Table 4).

In conclusion, methanol, water and chloroform extracts of *Rhus coriaria* L. showed anticancer effects on A549 and HT-29. It was determined that the chloroform extract of *Rhus coriaria* L. had the highest anticancer effect. Considering the studies in the literature regarding the anticancer activity of *Rhus coriaria* L., it has been shown that it inhibits the growth and proliferation of cancer cells in cervical cancer. Additionally, it has been reported that *Rhus coriaria* L. may be a therapeutic drug candidate in cervical cancer [41]. In another study on breast cancer, it was determined that the methanol extract of *Rhus coriaria* L. had an anticancer effect on MDA-MB-231 and MCF-7 [42]. In addition to MCF-7 cells, it has been reported that *Rhus coriaria* L. application inhibits the proliferation of cancer cells in PC-3 and SKOV3 cancer lines [15]. Based on the study findings and the literature, we can say that *Rhus coriaria* L. demonstrates anticancer activity on many cancer cells. Finally, the anticancer effect of *Rhus coriaria* L. ethanol extract on HT-29 and Caco-2 has been investigated in the literature. In this study, it was determined that the plant extract reduced cell viability and colony growth. In addition, it was shown that *Rhus coriaria* L. ethanol extract induced apoptosis via Caspase-7. It has been suggested that *Rhus coriaria* L. ethanol extract may have anticancer potential due to its anticancer effects on HT-29 and Caco-2 [43]. In our study, unlike the literature, we used A549 cancer cells in addition to HT-29 cells. However, unlike the literature, in our study we investigated the anticancer effects of *Rhus coriaria* L. methanol, water and chloroform extracts on A549, HT-29 cancer cells and normal fibroblast L929 cells. In light of the findings obtained from this study, we determined that *Rhus coriaria* L. extracts were selectively anticancer in A549 and HT-29 cancer cells.

### 3.3. Expression Levels of Apoptosis Genes

**Figure 4:**
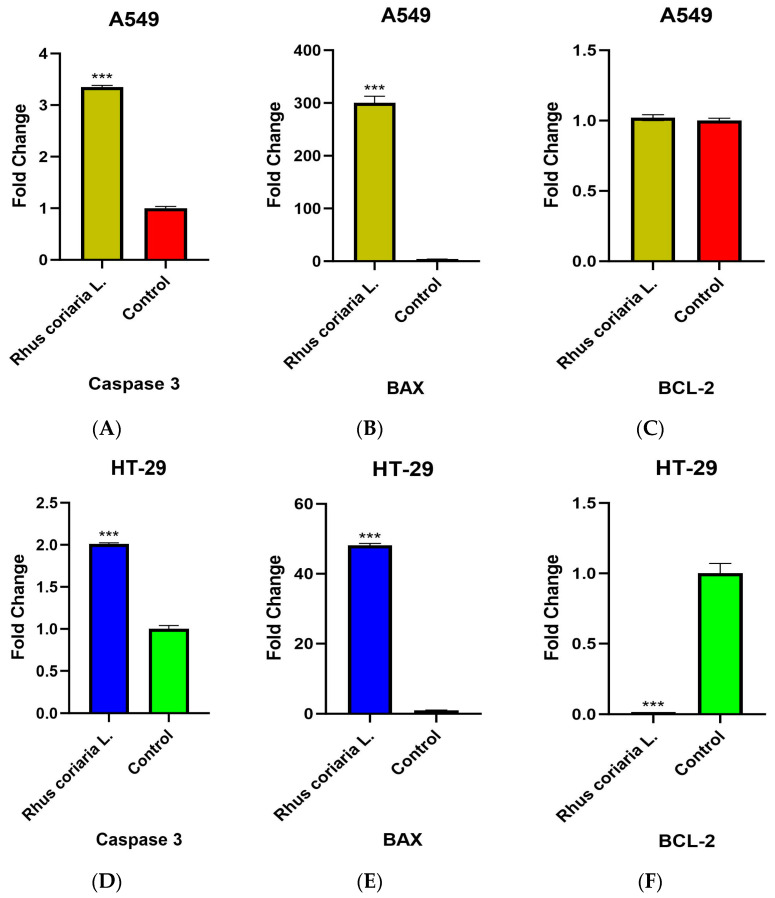
Expression profiles of apoptosis genes in (**A**–**C**) A549 and (**D**–**F**) HT-29 cells treated with IC_50_ dose of chloroform extract of *Rhus coriaria* L. Error bars represent standard deviation. (*** *p* < 0.001).

Caspase-3, BAX and BCL-2 expression levels were determined in cDNA samples obtained from A549 and HT-29 cell lines and A549 and HT-29 control group cell lines, to which the IC_50_ dose of chloroform extract of *Rhus coriaria* L. was applied for 24 h (Figure 4).

In A549 cell lines, BAX was determined to be increased by 300.33-fold and Caspase-3 was determined to be increased by 3.35-fold at 24 h (*p* < 0.05). In HT-29 cell lines, BAX was determined to be increased by 48.14-fold and Caspase-3 was determined to be increased by 2.02-fold; in addition, BCL-2 was determined to be decreased by 58.82-fold at 24 h (*p* < 0.05).

It has been shown that the expressions of BCL-2 and Caspase-3 are regulated by *Rhus coriaria* L. in MCF-7, PC-3 and SKOV3 cancer cells. It was determined that Caspase-3 was overexpressed and BCL-2 was underexpressed in *Rhus coriaria* L.-treated cells. It has been reported that cell proliferation is inhibited by inducing apoptosis in MCF-7, PC-3 and SKOV3 cancer cells through increased levels of Caspase-3 and decreased levels of BCL-2 [15]. In our study, it was determined that Caspase-3 as well as the apoptotic protein BAX were expressed at high levels in A549 and HT-29 after the application of *Rhus coriaria* L. chloroform extract. Additionally, BCL-2 was expressed at low levels in HT-29 cells. As a result, we can say that following the application of chloroform extract of *Rhus coriaria* L, it induces apoptosis in these cancer cell lines through increased Caspase-3 and BAX expressions in A549 and HT-29 cancer cells and decreased BCL-2 expression in HT-29 cells. Increased levels of apoptosis by regulating the expressions of apoptosis genes in A549 and HT-29 cancer lines may contribute to the anticancer activity of *Rhus coriaria* L.

### 3.4. Expression Levels of ADC, ODC and AGMAT Genes Associated with the Polyamine Pathway

**Figure 5:**
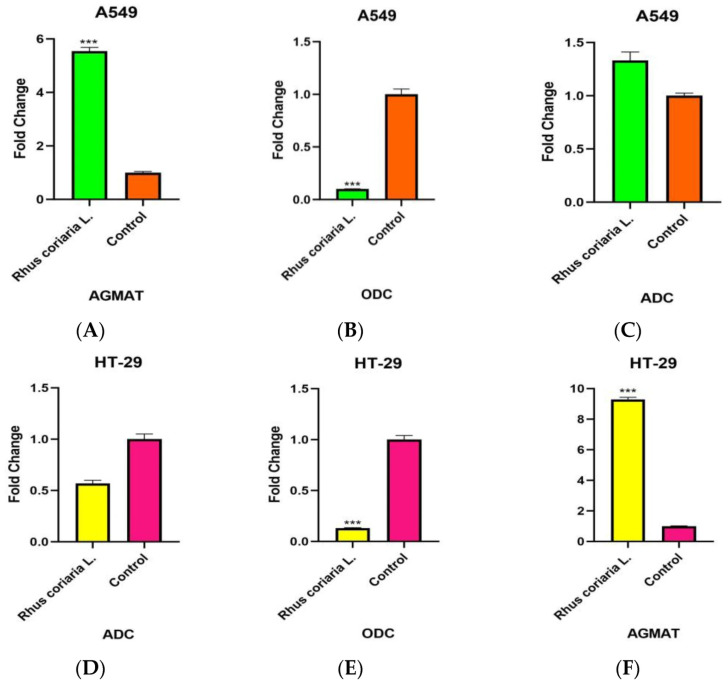
Expression profiles of polyamine pathway genes in (**A**–**C**) A549 and (**D**–**F**) HT-29 cells treated with IC_50_ dose of chloroform extract of *Rhus coriaria* L. Error bars represent standard deviation. (*** *p* < 0.001).

ADC, ODC and AGMAT expression levels were determined in cDNA samples obtained from A549 and HT-29 cell lines and A549 and HT-29 control group cell lines, to which the IC_50_ dose of chloroform extract of Rhus coriaria L. was applied for 24 h (Figure 5).

In A549 cell lines, it was determined that ODC decreased 9.90-fold and AGMAT increased 5.54-fold in 24 h (*p* < 0.05). In HT-29 cell lines, it was determined that ODC decreased 7.69-fold and AGMAT increased 9.29-fold in 24 h (*p* < 0.05).

There are no studies in the literature linking *Rhus coriaria* L. to the polyamine pathway. In our study, we found that ODC expression decreased and AGMAT expression increased in A549 and HT-29 cancer cells exposed to *Rhus coriaria* L. chloroform extracts. Since the decrease in ODC expression levels after exposure to *Rhus coriaria* L. extract can significantly inhibit polyamine synthesis, the increase in AGMAT expression levels in A549 and HT-29 tumor cells activated in response to this may be due to the defense mechanism of these tumor cells. In particular, the decrease in ODC expression levels may be mediating the decrease in polyamine levels in cancer cells. The decrease in polyamine levels can be associated with the decrease in the proliferation rates of cancer cells after exposure to plant extraction.

### 3.5. XO Enzyme Inhibition

**Figure 6:**
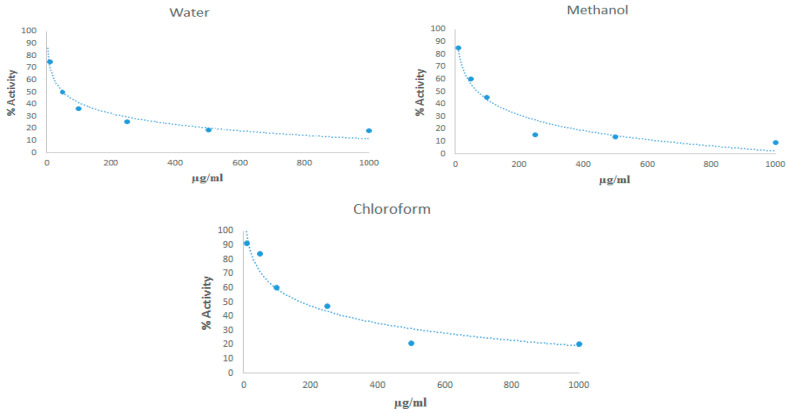
% Activation of XO enzyme at different doses of *Rhus coriaria* L. between 10 μg/mL and 1000 μg/mL.

The % activation of XO enzyme exposed to *Rhus coriaria* L. water, methanol and chloroform extract were determined (Figure 6).

The effects of extracts of *Rhus coriaria* L. against XO were evaluated by enzyme inhibition studies. The IC_50_ values of the water extract were determined as 49.65 μg/mL, the IC_50_ values of the methanol extract as 72.05 μg/mL and the IC_50_ values of the chloroform extract as 189.40 μg/mL. These findings indicate that among the extracts of *Rhus coriaria* L., the water extract was the one that inhibited the XO enzyme the most.

### 3.6. TAS Levels

TAS was determined in water, methanol and chloroform extract of *Rhus coriaria* L. between 10 µg/mL and 1000 µg/mL concentrations (Table 5).

It was determined that the TAS levels of extracts of *Rhus coriaria* L. increased with increasing concentration. The highest TAS level was at 1000 µg/mL concentrations. The TAS level of the water extract of *Rhus coriaria* L. at a concentration of 1000 µg/mL was 1.677 mmol Trolox Equiv./L, the TAS level at 1000 µg/mL concentration of methanol extract is 1.590 mmol Trolox Equiv./L and the TAS level of chloroform extract at a concentration of 1000 µg/mL was determined to be 1.078 mmol Trolox Equiv./L.

It has been reported that *Rhus coriaria* L. extracts have high antioxidant activity thanks to the antioxidant compounds they contain [44]. We determined that antioxidant status increased with increasing *Rhus coriaria* L. methanol, water and chloroform extract concentrations.

### 3.7. Theoretical Calculations

**Figure 7:**
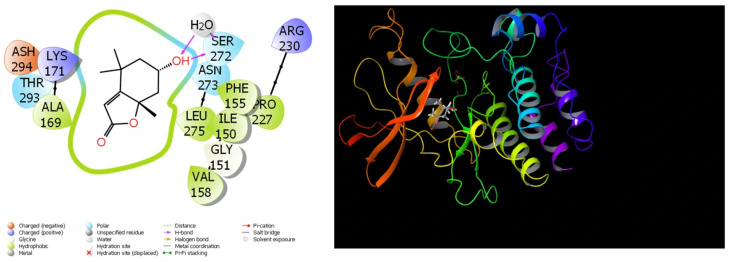
Presentation interactions of molecule (−)-Loliolide with colon cancer (PDB ID: 3DTC) protein.

**Figure 8:**
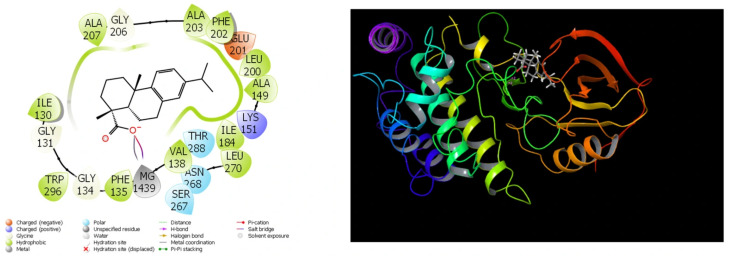
Presentation interactions of molecule dehydroabietic acid colon with cancer (PDB ID: 4UYA) protein.

**Figure 9:**
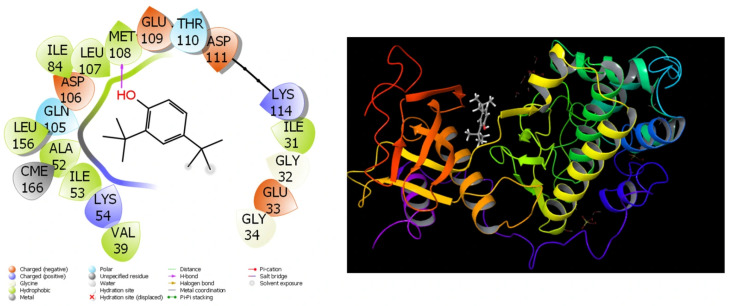
Presentation interactions of molecule phenol, 2,4-bis(1,1-dimethylethyl)-2 with lung cancer (PDB ID: 4ZXT) protein.

**Figure 10:**
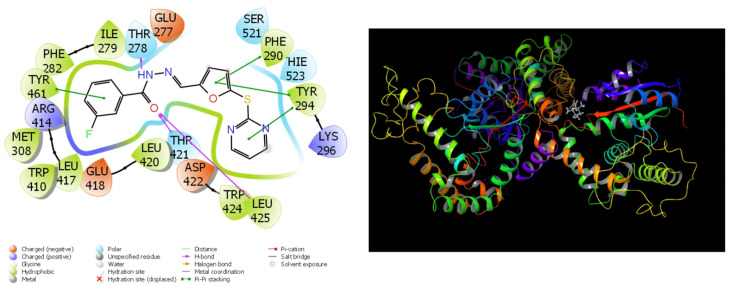
Presentation interactions of molecule 2(4H)-Benzofuranone, 5,6,7,7a-tetrahydro-4,4,7a-trimethyl- with lung cancer (PDB ID: 5ZMA) protein.

Because of the extensive use of theoretical research in recent studies, the comparisons of compounds’ biological actions have become much simpler and shorter. The completion of these calculations has simplified the process of identifying substances that are more effective and active prior to the initiation of experimental research. The results that have been obtained from the theoretical calculations are in close agreement with the results that have been obtained from the experimental data. A substantial quantity of parameters was created by these theoretical computations [45]. They are utilized in order to compare the numerical values of these parameters to the biological activities of molecules. For the purpose of molecular docking calculations, Table 6 and Figure 7, Figure 8, Figure 9 and Figure 10 have been provided.

Out of all the different measurements that are produced by molecular docking calculations, the docking score is the most important. This parameter provides a numerical representation of the interactions that take place between the protein and the chemical. The chemical that has the highest biological activity of the group is the one that has the lowest numerical value for this measurement. According to [46], the numerical value of this parameter diminishes as the interaction between molecules and proteins increases.

It should be common information that the interactions between chemicals and proteins in enzymes are the fundamental determinant of the biological activities of substances. These interactions exhibit a large number of interactions, including halogen, hydrogen bonds, π-π, polar and hydrophobic contacts, among others [47,48,49].

Among the parameters that are able to be determined in molecular docking simulations of molecules in *Rhus coriaria* L. extracts, the docking score and Glide Emodel parameters are the two that carry the greatest significance. The numerical values of these qualities provide information that is valuable in gaining an understanding of the biological activities of molecules [50]. The chemical that demonstrates the most biological activity against that protein is the one that has the lowest numerical values for the Glide Emodel parameter and the docking score [51]. The extensive interaction between the molecules and the proteins is demonstrated by the fact that the molecules exhibit a high degree of biological activity against the proteins. It ought to be general information that the molecule adheres to the protein more securely when there are more interactions between the molecule and the proteins, and this results in an increase in the molecule’s biological activity [52].

Glide ligand efficiency is yet another measurement that is obtained from calculations performed by molecular docking. It is a numerical depiction of the activity of *Rhus coriaria* L. extract against proteins. Glide Hbond is an additional statistic that indicates the number of hydrogen bonds that are formed over the course of interactions between proteins and molecules [50]. On the other hand, Glide EVW, which is a separate metric, is the quantity of Van der Waals interactions that occur in proteins between chemicals and proteins [51]. Glide Ecoul, which is a separate type of measure, is defined as the number of Coulomb interactions that occur within proteins between chemicals and proteins [52]. The numerical value that is obtained from the combination of several factors is the final parameter that is acquired from these computations; this value is known as Glide Einternal.

It was observed that the obtained Glide Score values followed a similar trend to other docking parameters such as Glide Emodel and binding energy. In particular, compounds exhibiting high biological activity potential had lower (more negative) values in both Glide Score and Glide Emodel, indicating that these compounds showed stronger binding behavior towards target proteins. This consistent trend increases the consistency of docking results and supports the idea that Glide Score is a reliable parameter for comparing the relative activities of compounds. Thus, the addition of Glide Score allowed for a clearer comparison of the binding profiles of molecules and strengthened the interpretability of docking analyses.

The binding free energy values of the molecule were estimated using the molecular mechanics generalized Born surface area (MM-GBSA) computations. The calculations yielded the finding that the dehydroabietic acid molecule was the one that had the docking score parameter with the 4UYA protein that was the lowest out of all the options. The energy values of the dehydroabietic acid molecule in relation to the 4UYA protein are presented in Table 7 as a result of this. The calculations demonstrate that the values of the binding free energy for the dehydroabietic acid molecule are equal to 16.68 among the 4UYA protein. Nevertheless, a significant number of interactions between the chemical and the protein were seen based on the values that were acquired. The interactions that are included in this list include the following: Coulomb, covalent, hydrogen bond, lipophilic, packing, SolvGB, and vdW interactions [21,52]. Lipo (lipophilic) and vdW (van der Waals) interactions, for instance, seem to have a larger negative value.

The chemicals discovered in *Rhus coriaria* L. extracts were compared in terms of their biological activities against different proteins. The chemicals with the highest activity were then analyzed by means of an ADME/T study. This analysis was carried out in order to theoretically predict the effects and responses of the molecules in human metabolism. A number of parameters were discovered as a result of this theoretical investigation, and they are shown in Table 8.

Molecular weight is the first of these measurements, as it necessitates a certain molecular weight for the molecule in question. Another statistic that might be taken into consideration is PISA, which is also known as solute total SASA. This value is the representation of the π (carbon and related hydrogen) component of SASA. An extra parameter is QP Polarizability, which is the expected polarizability in cubic angstroms.

Another important statistic is QPlogHERG, which is the numerical value of the estimated IC_50_ value of HERG K channels when they are blocked. The subsequent measurement is the permeability of Caco-2 cells at the gut–blood barrier for inactive transport, also known as QPPCaco. QPlogBB, which is an alternative measurement, is defined as the coefficient of the blood–brain barrier that the medication exhibits when it is administered orally. One, two or three are the three options for human oral absorption, which is a qualitative prediction that can be represented as low, medium or high oral absorption, respectively [21,51,52].

Of all the ADME/T characteristics, RuleOfFive and RuleOfThree are the two that are the most significant. The criteria RuleOfFive and RuleOfThree are of utmost importance. In order for these two parameter molecules to make a satisfactory pharmaceutical, the numerical value of the parameter is expected to be zero. The RuleOfFive parameter [53,54] is also known by the term Lipinski’s fifth Pfizer rule. The rules are as follows: Mol MW must be less than 500, QPlogP o/w must be less than 5, donorHB must be less than or equal to 5 and accptHB must be less than or equal to 10. Meanwhile, the third rule that Jorgensen devised is also known as the RuleOfThree parameter [55]. The three criteria are as follows: QPlogS must be greater than −5.7, QP PCaco must be greater than 22 nm/s and the number of primary metabolites must be less than 7 [49,50,51]. If the numerical value of the RuleOfThree parameter is equal to zero, this chemical can be ingested by mouth as a kind of treatment. The final and equally important parameter is Jm, which is the calculated maximal rate of transdermal transport represented as Kp × MW × S (μg cm–2h–1). QPlogKp and QPlogS are the origin of S and Kp, respectively, and they are the factors that determine the permeability of the skin and solubility in water. The numerical values of these parameters are calculated based on theoretical principles and are achieved by means of topical administration of pharmaceutical preparations or compounds that resemble drugs. The results of the calculations made it possible to examine the pharmacological features of the chemicals that were found in *Rhus coriaria* L. extracts and exhibited the highest levels of activity. It seemed that these chemicals had the potential to be developed into medications.

## 4. Conclusions

It has been determined that *Rhus coriaria* L. extract contains many compounds at different concentrations through the compounds found in its structure, inhibits the XO enzyme and has high antioxidant capacity. However, it was determined that it acts as an inducer of apoptosis by regulating the expression of apoptosis genes in A549 and HT-29 cell lines, and in this respect, it has anticancer activity. It has also been shown to regulate the expression of the polyamine pathway genes in A549 and HT-29 cell lines. In this way, we can say that it has an effect on cell proliferation via polyamine synthesis. Biological activities of chemicals found in *Rhus coriaria* L. extracts were compared against various proteins. Afterwards, the highest activity of these molecules is (−)-Loliolide molecule with 3DTC protein with −6.71, dehydroabietic acid molecule with 4UYA protein with −6.48 and phenol and 2,4-Bıs(1,1-Dimethylethyl)- with −6.48. It was observed that the molecule was with 4ZXT protein, and the molecule 2(4H)-Benzofuranone with −6.89, 5,6,7,7a-tetrahydro-4,4,7a-trimethyl- was found to be with 5ZMA protein. Afterwards, theoretical ADME/T analysis of these molecules was performed. As a result of these analyses, it was examined with many parameters. When the numerical values of the obtained parameters were examined, it was seen that they were an important guide for use in future studies.

## Figures and Tables

**Table 1:** Active constituents of *Rhus coriaria* L. chloroform extract.

Peak	Retention Time	Name of the Compound	Peak (%)
1	52.932	9-octadecenamide	4.76
2	55.978	Phenol, 3-pentadecyl-	29.48
3	56.585	Hexadecanoic acid	3.66
4	58.902	1-heptacosanol	20.73

**Table 2:** IC_50_ values of A549, HT-29 and L929 cells after 24 h exposure.

	A549 IC_50_	HT-29 IC_50_	L929 IC_50_
Water	136.10 μg/mL	603.10 μg/mL	1734.00 μg/mL
Methanol	116.80 μg/mL	307.20 μg/mL	1657.00 μg/mL
Chloroform	57.75 μg/mL	189.80 μg/mL	842.20 μg/mL

**Table 3:** IC50 values of A549, HT-29 and L929 cells after 48 h exposure.

	A549 IC_50_	HT-29 IC_50_	L929 IC_50_
Water	103.80 μg/mL	23.14 μg/mL	1035.00 μg/mL
Methanol	40.24 μg/mL	0.27 μg/mL	840.20 μg/mL
Chloroform	18.75 μg/mL	1.36 μg/mL	563.80 μg/mL

**Table 4:** IC_50_ values of A549, HT-29 and L929 cells after 72 h exposure.

	A549 IC_50_	HT-29 IC_50_	L929 IC_50_
Water	10.91 μg/mL	12.81 μg/mL	481.70 μg/mL
Methanol	5.47 μg/mL	11.78 μg/mL	411.70 μg/mL
Chloroform	2.55 μg/mL	24.00 μg/mL	303.00 μg/mL

**Table 5:** TAS levels of extracts of *Rhus coriaria* L.

	Water	Methanol	Chloroform
	TAS (mmol Trolox Equiv./L)	TAS (mmol Trolox Equiv./L)	TAS (mmol Trolox Equiv./L)
10 µg/mL	0.798 ± 0.022	0.881 ± 0.028	0.137 ± 0.011
100 µg/mL	1.577 ± 0.037	1.465 ± 0.039	0.571 ± 0.015
500 µg/mL	1.620 ± 0.043	1.576 ± 0.047	0.741 ± 0.021
1000 µg/mL	1.677 ± 0.024	1.590 ± 0.032	1.078 ± 0.033

**Table 6:** Numerical values of the docking parameters of molecule against protein.

**4ZXT**	**Docking Score**	**Glide Ligand Efficiency**	**Glide Hbond**	**Glide Evdw**	**Glide Ecoul**	**Glide Emodel**	**Glide** **Energy**	**Glide** **Einternal**	**Glide** **Posenum**
Phenol, 2,4-Bıs(1,1-Dımethylethyl)-	−6.48	−0.43	−0.32	−20.80	−5.66	−33.44	−26.45	4.52	333
(−)-Loliolide	−5.23	−0.37	−0.32	−21.85	−3.38	−33.04	−25.23	0.55	299
1-Heptacosanol	−2.69	−0.10	−0.53	−30.72	−5.26	−35.52	−35.99	12.38	86
2(4H)-Benzofuranone, 5,6,7,7a-tetrahydro-4,4,7a-trimethyl-	−4.42	−0.34	0.00	−21.65	0.13	−27.07	−21.52	0.00	387
2-Hexadecen-1-ol, 3,7,11,15-tetramethyl-	−1.01	−0.05	−0.27	−24.08	−6.80	−31.91	−30.88	2.13	44
2-Pentadecanone	0.50	0.03	−0.30	−23.94	−2.73	−22.08	−26.68	7.69	17
2-Pentadecanone, 6,10,14-trimethyl-	−0.68	−0.04	−0.16	−23.28	−3.01	−26.60	−26.29	1.82	160
2-Undecanone, 6,10-dimethyl-	−0.82	−0.06	−0.06	−20.75	−3.96	−25.03	−24.71	1.76	91
4,8,12,16-Tetramethylheptadecan-4-olide	−4.50	−0.20	−0.32	−27.75	−4.66	−39.46	−32.41	3.20	175
6,9-Octadecadienoic acid, methyl ester	0.05	0.00	−0.16	−27.89	−3.79	−28.25	−31.68	6.67	190
7,9-Di-tert-butyl-1-oxaspiro(4,5)deca-6,9-diene-2,8-dione	−3.93	−0.20	0.00	−23.88	−3.92	−34.53	−27.81	0.08	234
9,12-Octadecadienoic acid (Z,Z)-, methyl ester	−0.53	−0.03	−0.16	−27.09	−2.61	−27.92	−29.71	5.80	69
9,12-Octadecadienoyl chloride, (Z,Z)-	−0.76	−0.04	−0.16	−30.06	−3.27	−32.14	−33.33	5.64	77
9-Octadecenoic acid, methyl ester, (E)-	−0.06	0.00	−0.32	−27.63	−2.88	−27.61	−30.51	6.03	398
Behenic alcohol	−0.85	−0.04	−0.16	−24.31	−5.22	−24.87	−29.53	12.53	24
Bi-1-cycloocten-1-yl	−4.82	−0.30	0.00	−22.96	−0.02	−26.14	−22.98	3.02	293
Cembrene	−3.76	−0.19	0.00	−20.26	−1.96	−25.56	−22.22	2.55	236
Dec-2(E)-enal	2.36	0.21	−0.16	−16.01	−5.26	−15.37	−21.27	5.15	322
Decane (CAS)	−1.11	−0.11	0.00	−16.42	−0.13	−15.22	−16.55	4.68	326
Dehydroabıetıc Acıd	−5.14	−0.23	−0.24	−17.08	−3.22	−26.28	−20.31	2.71	168
Eicosanoic acid, 2,3-bis(acetyloxy)propyl ester	−2.49	−0.08	−0.32	−36.37	−7.53	−48.15	−43.90	5.81	222
Hept-2(E)-enal	−1.76	−0.22	−0.16	−11.91	−5.14	−17.97	−17.05	2.23	300
Heptadecanoic acid, 16-methyl-, methyl ester	−0.55	−0.03	−0.32	−26.86	−3.61	−28.42	−30.46	6.20	113
Heptadecyl alcohol	1.47	0.08	−0.32	−22.30	−6.70	−23.87	−29.01	4.84	159
Hexadecanamide	0.49	0.03	−0.45	−24.13	−4.67	−25.63	−28.79	4.20	270
Hexadecanoic acid, 1-[[[(2-aminoethoxy)hydroxyphosphinyl]oxy]methyl]-1,2-ethanediyl ester	−3.05	−0.06	−0.16	−36.20	−10.06	−48.86	−46.26	16.05	309
Hexadecanoic acid, 2-hydroxy-1-(hydroxymethyl)ethyl ester	−2.54	−0.11	−0.26	−24.41	−12.12	−39.71	−36.54	7.59	393
Methyleugenol	−4.51	−0.35	−0.25	−19.40	−5.37	−31.97	−24.76	0.49	150
Octadecanal	1.75	0.09	−0.16	−24.11	−4.68	−21.42	−28.79	8.17	54
Octadecanamide	0.13	0.01	−0.47	−27.13	−4.40	−28.37	−31.53	5.82	23
Performic acid, trimethylsilyl derivative	−3.66	−0.46	−0.16	−15.27	−4.04	−23.83	−19.31	0.43	265
Phenol, 3-pentadecyl-	−5.37	−0.24	−0.58	−30.43	−6.10	−46.23	−36.53	4.43	81
Podocarp-7-en-3-one, 13.beta.-methyl-13-vinyl-	−5.47	−0.26	0.00	−28.12	−1.56	−29.59	−29.69	2.06	390
Tetradecanal	2.11	0.14	0.00	−23.62	−3.31	−18.94	−26.93	8.81	166
Tricosane-2,4-dione	−2.51	−0.10	−0.48	−22.89	−9.09	−34.55	−31.98	5.49	25
Undecane	2.35	0.21	0.00	−17.81	−0.52	−14.80	−18.34	1.92	345
**5ZMA**	**Docking Score**	**Glide Ligand Efficiency**	**Glide Hbond**	**Glide Evdw**	**Glide Ecoul**	**Glide Emodel**	**Glide Energy**	**Glide Einternal**	**Glide Posenum**
(−)-Loliolide	−5.45	−0.39	−0.13	−19.59	−2.82	−27.89	−22.40	1.17	154
1-Heptacosanol	−3.02	−0.11	−0.32	−32.69	−6.66	−44.33	−39.35	5.13	324
2(4H)-Benzofuranone, 5,6,7,7a-tetrahydro-4,4,7a-trimethyl-	−6.89	−0.53	−0.08	−19.26	−3.60	−33.43	−22.86	0.00	326
2-Hexadecen-1-ol, 3,7,11,15-tetramethyl-	−2.88	−0.14	−0.32	−30.48	−1.99	−29.27	−32.47	13.50	101
2-Pentadecanone	−0.71	−0.04	−0.25	−26.09	−4.24	−27.90	−30.33	7.26	154
2-Pentadecanone, 6,10,14-trimethyl-	−2.41	−0.13	−0.33	−28.31	−4.33	−34.93	−32.64	4.23	252
2-Undecanone, 6,10-dimethyl-	−2.30	−0.16	−0.41	−23.82	−7.62	−31.19	−31.44	9.53	171
4,8,12,16-Tetramethylheptadecan-4-olide	−5.33	−0.23	0.00	−20.83	−2.31	−23.40	−23.15	7.64	135
5zma—minimized 2(4H)-Benzofuranone, 5,6,7,7a-tetrahydro-4,4,7a-trimethyl-	−6.89	−0.53	−0.08	−19.26	−3.60	−33.43	−22.86	0.00	326
6,9-Octadecadienoic acid, methyl ester	−2.18	−0.10	−0.16	−32.87	−4.06	−37.15	−36.93	8.17	315
7,9-Di-tert-butyl-1-oxaspiro(4,5)deca-6,9-diene-2,8-dione	−6.06	−0.30	0.00	−11.67	−1.75	−15.93	−13.42	10.10	20
9,12-Octadecadienoic acid (Z,Z)-, methyl ester	0.56	0.03	−0.26	−35.68	−3.01	−23.74	−38.69	10.82	161
9,12-Octadecadienoyl chloride, (Z,Z)-	−2.46	−0.12	0.00	−27.12	−0.89	−28.07	−28.01	6.39	396
9-Octadecenoic acid, methyl ester, (E)-	−1.66	−0.08	−0.16	−31.90	−4.02	−35.16	−35.93	8.47	188
Behenic alcohol	−3.98	−0.17	−0.16	−33.85	−3.10	−40.02	−36.95	11.74	352
Bi-1-cycloocten-1-yl	−4.27	−0.27	0.00	−26.04	0.40	−31.03	−25.64	2.69	120
Cembrene	−4.82	−0.24	0.00	−23.64	0.70	−27.66	−22.94	0.04	152
Dec-2(E)-enal	1.34	0.12	0.00	−20.58	−3.44	−20.56	−24.01	2.71	128
Decane (CAS)	−0.98	−0.10	0.00	−18.63	−0.57	−18.39	−19.20	3.90	178
DEHYDROABIETIC ACID	−5.69	−0.26	0.00	−24.40	2.58	−20.87	−21.82	2.60	57
Eicosanoic acid, 2,3-bis(acetyloxy)propyl ester	−3.50	−0.11	0.00	−43.34	−2.10	−50.71	−45.43	9.45	284
Hept-2(E)-enal	−2.91	−0.36	−0.19	−17.53	−4.18	−23.35	−21.70	4.67	70
Heptadecanoic acid, 16-methyl-, methyl ester	−1.72	−0.08	0.00	−31.96	−2.64	−34.94	−34.60	5.06	76
Heptadecyl alcohol	0.73	0.04	−0.16	−26.42	−6.08	−28.28	−32.51	5.50	75
Hexadecanamide	−1.06	−0.06	−0.13	−29.66	−5.19	−32.99	−34.85	7.66	50
Hexadecanoic acid, 1-[[[(2-aminoethoxy)hydroxyphosphinyl]oxy]methyl]-1,2-ethanediyl ester	−0.43	−0.01	−0.32	−37.19	−3.58	−41.67	−40.77	12.71	166
Hexadecanoic acid, 2-hydroxy-1-(hydroxymethyl)ethyl ester	−4.52	−0.20	0.00	−31.66	−5.71	−41.94	−37.37	11.60	37
Methyleugenol	−5.96	−0.46	0.00	−28.10	−0.44	−39.05	−28.54	0.40	305
Neophytadiene	−1.39	−0.07	0.00	−29.76	−0.30	−27.98	−30.06	8.15	236
Octadecanal	−0.89	−0.05	−0.29	−26.04	−3.43	−26.79	−29.47	8.16	86
Octadecanamide	−1.81	−0.09	−0.16	−29.23	−3.43	−31.07	−32.65	10.40	357
Performic acid, trimethylsilyl derivative	−4.05	−0.51	−0.10	−18.29	−3.74	−27.41	−22.03	0.26	24
PHENOL, 2,4-BIS(1,1-DIMETHYLETHYL)-	−5.46	−0.36	0.00	−21.87	−1.26	−28.55	−23.13	1.66	398
Phenol, 3-pentadecyl-	−5.97	−0.27	−0.21	−36.61	−1.04	−48.23	−37.65	8.26	288
Podocarp-7-en-3-one, 13.beta.-methyl-13-vinyl-	−5.55	−0.26	0.00	−18.27	−1.95	−36.04	−20.22	2.17	265
Tetradecanal	−0.26	−0.02	−0.38	−23.69	−5.75	−26.37	−29.44	7.11	162
Tricosane-2,4-dione	−5.14	−0.21	−0.16	−40.58	−4.21	−54.05	−44.79	9.13	153
Undecane	1.58	0.14	0.00	−21.08	0.42	−17.29	−20.66	3.27	13
**3DTC**	**Docking Score**	**Glide Ligand Efficiency**	**Glide Hbond**	**Glide Evdw**	**Glide Ecoul**	**Glide Emodel**	**Glide Energy**	**Glide Einternal**	**Glide Posenum**
(−)-Loliolide	−6.71	−0.48	−0.55	−13.26	−7.58	−28.66	−20.85	1.23	41
1-Heptacosanol	−2.86	−0.10	−0.32	−36.22	−3.69	−42.85	−39.92	7.52	286
2(4H)-Benzofuranone, 5,6,7,7a-tetrahydro-4,4,7a-trimethyl-	−5.55	−0.43	0.00	−18.11	−1.66	−20.99	−19.77	0.00	184
2-Hexadecen-1-ol, 3,7,11,15-tetramethyl-	−4.01	−0.19	−0.51	−33.25	−5.08	−44.49	−38.33	2.89	203
2-Pentadecanone	−1.42	−0.09	−0.29	−28.73	−2.68	−31.77	−31.40	4.68	185
2-Pentadecanone, 6,10,14-trimethyl-	−3.63	−0.19	−0.32	−30.06	−2.14	−35.14	−32.20	7.80	271
2-Undecanone, 6,10-dimethyl-	−2.63	−0.19	−0.32	−23.40	−2.54	−28.81	−25.94	2.80	136
3DTC—minimized (-)-Loliolide	−6.71	−0.48	−0.55	−13.26	−7.58	−28.66	−20.85	1.23	41
4,8,12,16-Tetramethylheptadecan-4-olide	−5.44	−0.24	0.00	−32.00	−1.27	−40.94	−33.28	7.12	174
6,9-Octadecadienoic acid, methyl ester	−2.23	−0.11	0.00	−38.11	0.35	−39.91	−37.76	6.24	87
7,9-Di-tert-butyl-1-oxaspiro(4,5)deca-6,9-diene-2,8-dione	−6.17	−0.31	0.00	−11.60	−0.51	−23.87	−12.11	6.86	131
9,12-Octadecadienoic acid (Z,Z)-, methyl ester	−2.08	−0.10	0.00	−38.20	−1.81	−41.38	−40.01	6.65	364
9,12-Octadecadienoyl chloride, (Z,Z)-	−2.09	−0.10	0.00	−36.52	0.38	−38.39	−36.13	4.81	257
9-Octadecenoic acid, methyl ester, (E)-	−2.10	−0.10	−0.08	−38.64	−2.28	−42.83	−40.93	6.63	287
Behenic alcohol	−3.45	−0.15	−0.03	−35.95	−1.93	−42.08	−37.88	7.69	290
Bi-1-cycloocten-1-yl	−6.37	−0.40	0.00	−21.00	−0.24	−22.52	−21.24	2.96	167
Cembrene	−6.32	−0.32	0.00	−12.42	−0.16	−42.99	−12.57	2.62	168
Dec-2(E)-enal	0.29	0.03	−0.32	−21.77	−2.39	−22.52	−24.16	2.45	54
Decane (CAS)	−1.89	−0.19	0.00	−18.95	0.08	−18.29	−18.87	4.97	339
DEHYDROABIETIC ACID	−4.18	−0.19	0.00	−9.17	0.47	−15.76	−8.69	2.11	340
Eicosanoic acid, 2,3-bis(acetyloxy)propyl ester	−5.12	−0.16	−0.04	−44.76	−3.06	−58.99	−47.81	7.14	129
Hept-2(E)-enal	−3.38	−0.42	−0.32	−15.40	−3.15	−22.14	−18.55	0.95	214
Heptadecanoic acid, 16-methyl-, methyl ester	−2.33	−0.11	−0.32	−36.97	−3.15	−41.69	−40.11	7.09	380
Heptadecyl alcohol	−0.28	−0.02	−0.32	−29.54	−3.56	−30.42	−33.10	6.44	142
Hexadecanamide	−1.02	−0.06	−0.32	−31.82	−4.21	−36.49	−36.03	3.78	244
Hexadecanoic acid, 1-[[[(2-aminoethoxy)hydroxyphosphinyl]oxy]methyl]-1,2-ethanediyl ester	−5.48	−0.12	−0.23	−50.89	−2.72	−65.28	−53.61	19.10	269
Hexadecanoic acid, 2-hydroxy-1-(hydroxymethyl)ethyl ester	−4.41	−0.19	0.00	−35.59	−2.44	−45.09	−38.03	9.29	192
Methyleugenol	−5.43	−0.42	0.00	−24.52	−1.46	−34.16	−25.98	1.31	228
Neophytadiene	−1.97	−0.10	0.00	−34.87	0.44	−36.35	−34.43	3.79	267
Octadecanal	−1.10	−0.06	−0.04	−34.53	−1.54	−36.92	−36.07	2.93	365
Octadecanamide	−1.80	−0.09	−0.34	−34.01	−4.57	−40.95	−38.59	3.85	114
Performic acid, trimethylsilyl derivative	−3.33	−0.42	0.00	−15.48	−0.17	−18.74	−15.65	0.37	325
PHENOL, 2,4-BIS(1,1-DIMETHYLETHYL)-	−6.48	−0.43	0.00	−21.02	−0.40	−26.40	−21.42	4.24	184
Phenol, 3-pentadecyl-	−5.09	−0.23	0.00	−39.01	−2.35	−51.69	−41.36	2.97	289
Tetradecanal	0.15	0.01	−0.02	−29.32	−1.16	−29.38	−30.48	1.67	120
Tricosane-2,4-dione	−5.14	−0.21	−0.32	−40.21	−3.13	−55.85	−43.34	4.90	264
Undecane	1.83	0.17	0.00	−20.22	−0.29	−16.98	−20.51	2.29	283
**4UYA**	**Docking Score**	**Glide Ligand Efficiency**	**Glide Hbond**	**Glide Evdw**	**Glide Ecoul**	**Glide Emodel**	**Glide Energy**	**Glide Einternal**	**Glide Posenum**
DEHYDROABIETIC ACID	−9.64	−0.44	0.00	−22.05	−13.40	−82.65	−35.46	3.73	334
(−)-Loliolide	−5.55	−0.40	0.00	−16.69	−13.94	−44.26	−30.63	0.35	136
1-Heptacosanol	−4.04	−0.14	−0.32	−28.67	−5.36	−35.62	−34.03	12.26	83
2(4H)-Benzofuranone, 5,6,7,7a-tetrahydro-4,4,7a-trimethyl-	−5.20	−0.40	0.00	−16.71	−10.69	−38.64	−27.41	0.00	279
2-Hexadecen-1-ol, 3,7,11,15-tetramethyl-	−2.78	−0.13	−0.05	−28.18	−14.82	−49.48	−43.00	6.83	120
2-Pentadecanone	−1.11	−0.07	0.00	−26.34	−11.67	−37.42	−38.01	7.65	210
2-Pentadecanone, 6,10,14-trimethyl-	−2.53	−0.13	0.00	−28.05	−10.88	−44.46	−38.93	3.98	400
2-Undecanone, 6,10-dimethyl-	−2.23	−0.16	0.00	−20.79	−11.71	−36.17	−32.51	3.90	56
4,8,12,16-Tetramethylheptadecan-4-olide	−6.07	−0.26	0.00	−30.16	−14.84	−61.91	−45.00	11.65	110
6,9-Octadecadienoic acid, methyl ester	−2.49	−0.12	0.00	−28.27	−11.96	−44.69	−40.23	6.51	108
7,9-Di-tert-butyl-1-oxaspiro(4,5)deca-6,9-diene-2,8-dione	−5.29	−0.26	0.00	−22.04	−14.75	−53.66	−36.79	0.02	368
9,12-Octadecadienoic acid (Z,Z)-, methyl ester	−2.08	−0.10	−0.16	−26.58	−10.12	−41.43	−36.70	4.09	325
9,12-Octadecadienoyl chloride, (Z,Z)-	−2.30	−0.11	−0.06	−33.50	−8.34	−46.19	−41.84	5.11	366
9-Octadecenoic acid, methyl ester, (E)-	−2.06	−0.10	−0.18	−30.69	−9.52	−45.39	−40.21	2.97	154
Behenic alcohol	−4.22	−0.18	0.00	−28.70	−12.63	−46.60	−41.33	13.39	150
Bi-1-cycloocten-1-yl	−5.10	−0.32	0.00	−20.12	0.26	−24.69	−19.86	0.04	19
Cembrene	−5.22	−0.26	0.00	−27.41	−0.45	−31.60	−27.86	3.30	91
Dec-2(E)-enal	0.36	0.03	0.00	−16.74	−11.49	−25.65	−28.24	3.47	237
Decane (CAS)	−1.00	−0.10	0.00	−11.49	0.57	−6.61	−10.92	9.07	384
DEHYDROABIETIC ACID	−9.58	−0.44	−0.01	−18.33	−19.23	−91.27	−37.56	2.32	154
Eicosanoic acid, 2,3-bis(acetyloxy)propyl ester	−4.26	−0.13	−0.25	−38.19	−10.92	−60.49	−49.11	5.55	302
Hept-2(E)-enal	−2.59	−0.32	0.00	−10.85	−11.94	−28.26	−22.80	0.01	135
Heptadecanoic acid, 16-methyl-, methyl ester	−1.25	−0.06	−0.05	−31.50	−10.00	−41.76	−41.50	7.60	328
Heptadecyl alcohol	0.12	0.01	0.00	−22.69	−13.26	−32.45	−35.95	6.31	193
Hexadecanamide	−1.76	−0.10	0.00	−27.77	−13.48	−44.31	−41.26	5.41	119
Hexadecanoic acid, 1-[[[(2-aminoethoxy)hydroxyphosphinyl]oxy]methyl]-1,2-ethanediyl ester	−8.00	−0.17	−0.38	−29.96	−30.28	−108.66	−60.25	23.38	60
Hexadecanoic acid, 2-hydroxy-1-(hydroxymethyl)ethyl ester	−6.37	−0.28	0.00	−24.42	−26.37	−68.28	−50.79	18.35	306
Methyleugenol	−4.70	−0.36	−0.01	−18.82	−14.23	−46.71	−33.06	0.53	388
Neophytadiene	−0.96	−0.05	0.00	−28.01	−0.23	−26.04	−28.24	7.87	281
Octadecanal	−1.16	−0.06	−0.02	−27.98	−10.67	−39.41	−38.64	4.93	46
Octadecanamide	−2.43	−0.12	−0.15	−31.80	−14.28	−50.06	−46.08	8.03	72
Performic acid, trimethylsilyl derivative	−4.64	−0.58	−0.05	−15.25	−14.70	−40.76	−29.94	2.57	369
PHENOL, 2,4-BIS(1,1-DIMETHYLETHYL)-	−5.11	−0.34	0.00	−15.63	−11.45	−37.77	−27.08	2.26	214
Phenol, 3-pentadecyl-	−4.53	−0.21	0.00	−26.27	−13.20	−51.35	−39.47	4.53	32
Podocarp-7-en-3-one, 13.beta.-methyl-13-vinyl-	−6.25	−0.30	0.00	−25.21	−11.28	−51.06	−36.49	1.60	389
Tetradecanal	−0.38	−0.03	0.00	−22.73	−12.74	−34.15	−35.47	4.81	104
Tricosane-2,4-dione	−5.83	−0.23	−0.04	−29.11	−20.47	−69.65	−49.58	5.35	374
Undecane	1.19	0.11	0.00	−20.69	0.11	−17.84	−20.58	2.63	64

**Table 7:** MM-GBSA parameter of dehydroabietic acid with 4UYA protein.

MMGBSA dG Bind	16.68
MMGBSA dG Bind Coulomb	−15.61
MMGBSA dG Bind Covalent	0.79
MMGBSA dG Bind Hbond	−0.03
MMGBSA dG Bind Lipo	−8.62
MMGBSA dG Bind Packing	0.00
MMGBSA dG Bind Solv GB	60.57
MMGBSA dG Bind vdW	−20.43

**Table 8:** ADME properties of molecules. * concern below −5, ** <25 is poor and >500 is great, *** <25% is poor and >80% is high.

	(−)-Loliolide	Dehydroabietic Acid	Phenol, 2,4-bis(1,1-dimethylethyl)-2	2(4H)-Benzofuranone, 5,6,7,7a-tetrahydro-4,4,7a-trimethyl-	Referance Range
mol_MW	196	300	206	180	130–725
dipole (D)	7.9	2.3	1.4	6.9	1.0–12.5
SASA	388	562	463	385	300–1000
FOSA	264	426	364	292	0–750
FISA	100	73	30	68	7–330
PISA	24	64	69	25	0–450
WPSA	0	0	0	0	0–175
volume (A^3^)	665	1020	808	650	500–2000
donorHB	1	1	1	0	0–6
accptHB	4.7	2	0.75	3	2.0–20.0
glob (Sphere = 1)	0.9	0.9	0.9	0.9	0.75–0.95
QPpolrz (A^3^)	20.0	34.0	24.9	20.1	13.0–70.0
QPlogPC16	6.0	9.0	6.9	5.4	4.0–18.0
QPlogPoct	11.3	13.3	9.2	8.5	8.0–35.0
QPlogPw	7.4	5.0	3.0	4.2	4.0–45.0
QPlogPo/w	0.9	4.9	3.8	1.6	−2.0–6.5
QPlogS	−1.8	−5.5	−3.8	−1.9	−6.5–0.5
CIQPlogS	−1.7	−4.8	−3.3	−1.7	−6.5–0.5
QPlogHERG	−2.5	−2.1	−3.4	−2.6	*
QPPCaco (nm/s)	1118	514	5122	2245	**
QPlogBB	−0.2	−0.2	0.1	0.1	−3.0–1.2
QPPMDCK (nm/s)	558	306	2892	1186	**
QPlogKp	−3.2	−2.4	−1.5	−2.7	Kp in cm/hr
IP (ev)	10.6	9.2	9.0	10.6	7.9–10.5
EA (eV)	0.3	−0.4	−0.3	0.3	−0.9–1.7
#metab	1	2	2	0	1–8
QPlogKhsa	−0.4	0.8	0.5	−0.3	−1.5–1.5
Human Oral Absorption	3	3	3	3	-
Percent Human Oral Absorption	87	100	100	96	***
PSA	59	42	17	41	7–200
RuleOfFive	0	0	0	0	Maximum is 4
RuleOfThree	0	0	0	0	Maximum is 3
Jm	2.3	0.0	0.8	4.3	**-**

## Data Availability

The original contributions presented in this study are included in the article. Further inquiries can be directed to the corresponding authors.
